# Association of prognostic nutritional index with fecal incontinence and fecal incontinence severity index in individuals with osteoporosis: mediating roles of C-reactive protein and gamma-glutamyl transferase

**DOI:** 10.3389/fnut.2026.1828358

**Published:** 2026-06-09

**Authors:** Feng Li, Jianbin Chen, Longxin Zhou, Chaoran Yang, Zhenxing Zhu

**Affiliations:** Department of Rehabilitation Medicine, Ganzhou People’s Hospital, Ganzhou, Jiangxi, China

**Keywords:** C-reactive protein, fecal incontinence, fecal incontinence severity index, gamma-glutamyl transferase, osteoporosis, prognostic nutritional index

## Abstract

**Background:**

Malnutrition is increasingly recognized as a contributor to both osteoporosis and gastrointestinal dysfunction. The prognostic nutritional index, derived from serum albumin and lymphocyte counts, reflects nutritional and immune status. However, its association with fecal incontinence in osteoporotic patients remains unexplored.

**Methods:**

This cross-sectional study included osteoporotic patients from one Chinese tertiary hospital between 2021 and 2025. FI was assessed using the bowel health questionnaire, and severity was quantified by the fecal incontinence severity index (FISI). Multivariable logistic and linear regression models examined associations between PNI, FI, and FISI in osteoporotic patients. Restricted cubic splines assessed dose–response relationships. ROC curves assessed the predictive effect of PNI on fecal incontinence in individuals with osteoporosis. Mediation analyses evaluated the roles of C-reactive protein (CRP) and gamma-glutamyl transferase (GGT).

**Results:**

Among 10,906 osteoporotic patients, FI prevalence was 8.78%. PNI was independently and negatively associated with FI (OR = 0.85; 95% CI: 0.76–0.93) and FISI (*β* = −0.46; 95% CI: −0.57 to −0.38), with linear dose–response relationships (*P* for nonlinear > 0.05). PNI demonstrated moderate predictive performance for FI (AUC = 0.723, 95% CI: 0.707–0.739), outperforming alternative malnutrition indices, and it also increased the discriminative predictive capacity (NRI = 0.13, 95% CI: 0.06–0.22; IDI = 0.08, 95% CI: 0.03–0.14). In cross-sectional exploratory mediation analysis, CRP and GGT were associated with these relationships, accounting for 24.2–29.0% and 11.6–17.5% of the total statistical associations, respectively. In addition, the association also remained consistent across subgroups and sensitivity analyses.

**Conclusion:**

The PNI is independently and linearly associated with FI and its severity in osteoporotic patients, partially mediated by inflammation and oxidative stress. PNI may serve as a useful nutritional indicator to identify at-risk individuals for targeted intervention.

## 1 Introduction

Osteoporosis is a systemic skeletal disease characterized by low bone mass and deterioration of the microarchitecture of bone tissue, leading to increased bone fragility and a consequent increase in fracture risk (1). Osteoporosis represents a major global health burden, affecting more than 200 million individuals worldwide. The condition is responsible for an estimated 8.9 million fractures annually, corresponding to one osteoporotic fracture every 3 s (2). It is projected that approximately one in three women and one in five men aged 50 years or older will experience an osteoporotic fracture during their remaining lifetime (2). Therefore, the adverse health consequences of osteoporosis warrant close attention. Some studies have demonstrated that osteoporosis confers significant morbidity and mortality primarily through its association with fragility fractures, which is largely attributable to complications of prolonged immobilization, such as venous thromboembolism, pneumonia, pressure ulcers, and muscle wasting (3). Additionally, gastrointestinal symptoms, including constipation and fecal incontinence, are frequently observed in patients with advanced osteoporosis (4). These symptoms are attributable to a confluence of biomechanical, iatrogenic, and comorbid factors: elevated intra-abdominal pressure induced by osteoporotic vertebral fractures, as well as the use of pharmacotherapies for osteoporosis, such as calcium supplements, bisphosphonates, and analgesics, particularly opioids, which are well-established contributors to gastrointestinal dysmotility and constipation (5). Evidence supports that compared with osteoporosis alone, osteoporosis complicated by comorbidities is associated with a significantly poorer prognosis and higher mortality rate (6). Further research using large-scale cohort studies is warranted to clarify the adverse health outcomes and potential risk factors associated with complications of osteoporosis to improve patient prognosis.

Fecal incontinence (FI), as a bowel symptom, may be one of the complications of osteoporosis (7). It is defined as the recurrent, uncontrolled passage of solid or liquid stool or flatus through the anus for a duration of at least 1 month (7). The condition encompasses a spectrum of clinical presentations, including passive incontinence (discharge without awareness), urge incontinence (inability to reach the toilet in time despite warning), and seepage often associated with constipation (8). It results from a multifactorial disruption of the complex mechanisms maintaining continence, which may involve dysfunction of the anal sphincters, pelvic floor musculature, or sacral nerve innervation (9). In patients with advanced osteoporosis, vertebral compression fractures often lead to progressive kyphosis and subsequent reduction in abdominal volume, resulting in chronically elevated intra-abdominal pressure that can overwhelm pelvic floor supportive mechanisms, predisposing individuals to stress-related fecal leakage (10). Currently, there are limited studies on potential risk factors of FI in patients with osteoporosis.

Malnutrition plays a key role in the development and progression of various diseases, which is defined as a state resulting from a lack of intake or uptake of energy, protein, or other nutrients that leads to an adverse effect on body composition, function, and clinical outcomes (11). Epidemiological studies indicate that malnutrition increases the risk of developing isolated FI and osteoporosis (12). However, the role of malnutrition in the combined occurrence of osteoporosis and FI remains unclear. The prognostic nutritional index (PNI) is regarded as a malnutrition indicator, calculated based on serum albumin concentration and peripheral blood lymphocyte count, and offers several distinct advantages over conventional nutritional assessment tools in clinical practice (13, 14). Unlike subjective screening methods such as the subjective global assessment (SGA), PNI provides a fully objective and quantifiable measure derived from routine laboratory data, ensuring high reproducibility and minimizing evaluator bias. By integrating serum albumin, as a marker of visceral protein reserve, with total lymphocyte count, as a surrogate for immune competence, PNI uniquely captures the dual dimensions of nutritional depletion and immunosuppression (15, 16). This composite pattern endows PNI with superior prognostic stratification capacity across diverse disease settings, where it independently predicts postoperative complications, infection risk, and long-term survival. Furthermore, its simplicity, low cost, and reliance on widely available blood tests make it particularly suitable for large-scale epidemiological studies and routine clinical application in resource-limited settings (17). However, there are no studies on the association of PNI with FI in patients with osteoporosis and potential biomechanisms.

To address the above-mentioned research gaps, we conducted a cross-sectional study to explore the association between PNI and FI in patients with osteoporosis. This study could validate PNI as a practical tool for identifying high-risk individuals who may benefit from targeted nutritional support, thereby offering a potential pathway to reduce FI prevalence and improve overall prognosis in the osteoporotic population.

## 2 Methods

### 2.1 Study design

A retrospective cross-sectional analysis was conducted to investigate the relationship between the PNI and fecal incontinence (FI) among individuals diagnosed with osteoporosis. Data were derived from health examination records from the rehabilitation medicine center of Ganzhou People’s Hospital between January 1, 2021, and January 1, 2025. All participants were community-dwelling adults who underwent routine annual health examinations at the center. Individuals were not recruited from outpatient clinics, inpatient wards, or follow-up cohorts. To ensure the uniqueness and consecutively of participants’ data, we retained only the first eligible visit for each individual during the study period. In addition, all individuals who met the eligibility criteria during the study period were enrolled to make these participants be consecutive cases. Furthermore, duplicate individuals were identified and excluded using unique hospital medical record numbers; for patients with multiple records, only the earliest visit was kept, and subsequent visits were removed. The research protocol received ethical approval from the institutional review boards of participating hospitals (GZPH-2025-28). All participants provided written informed consent before enrollment, in accordance with the ethical standards outlined in the Declaration of Helsinki.

**Figure 1:**
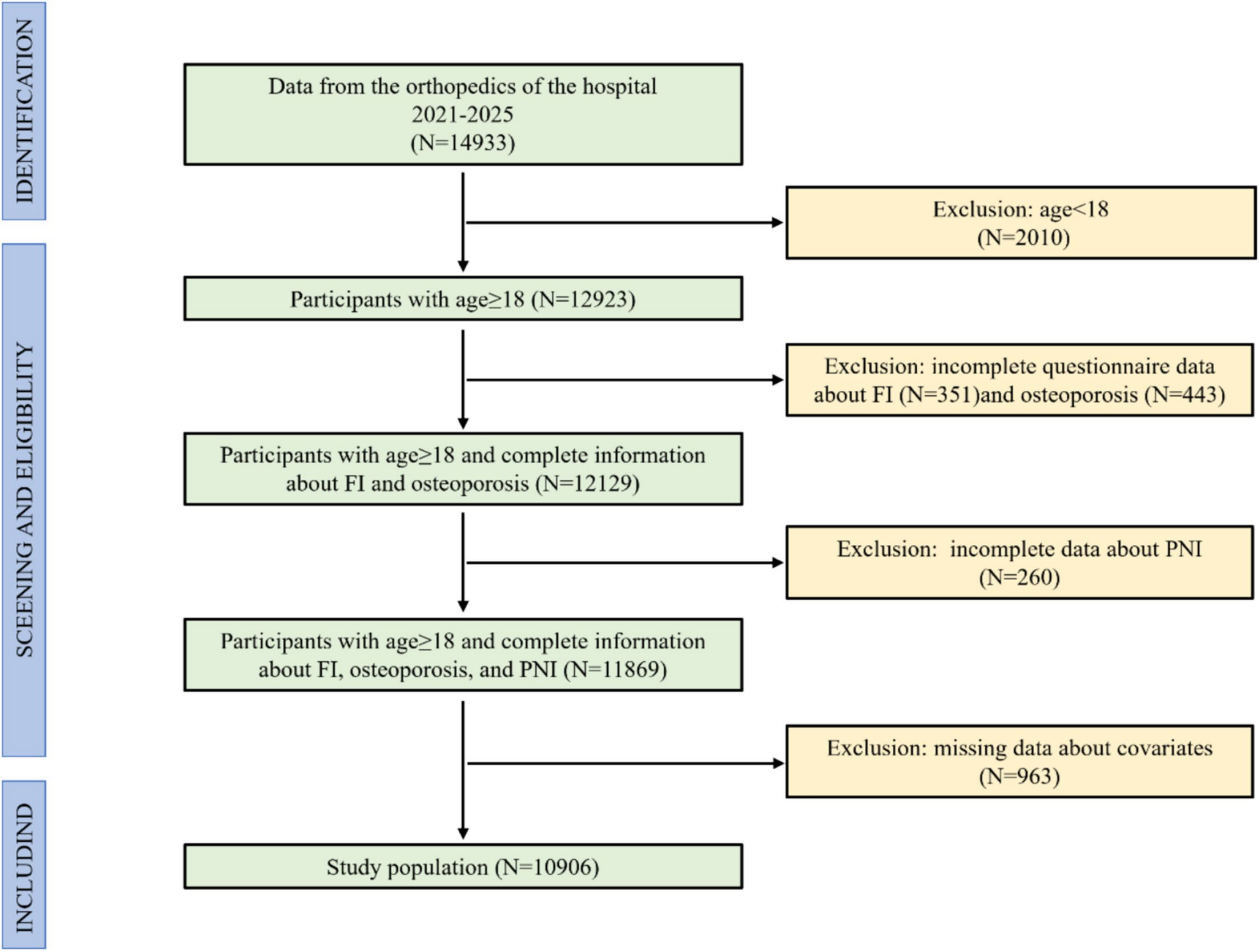
Flow chart of participants in inclusion and exclusion criteria.

In this study, of the initial 14,933 participants, 2010 were excluded for being younger than 18 years. Among the remaining individuals, further exclusions were made due to missing data: 351 for FI, 443 for osteoporosis, 260 for PNI, and 963 for covariates. After these sequential exclusions, the final analytic cohort comprised 10,906 osteoporosis patients aged 18 years or older with complete information on all study variables. A detailed flowchart of the participant selection process is provided in Figure 1.

### 2.2 Calculation of PNI, CONUT, GNRI

The calculation formula for PNI is as follows: PNI = serum albumin (g/L) + 5 × total peripheral blood lymphocyte count (×10^9^/L) (18). PNI values were interpreted as follows: >50 indicated normal nutritional status; 45–50, mild malnutrition; 40–45, moderate malnutrition; and <40, severe malnutrition (18). Controlling nutritional status (CONUT) was calculated from serum albumin, total cholesterol, and total lymphocyte count. Albumin scores were assigned as 0 points for ≥3.5 g/dL, 2 points for 3.0–3.4 g/dL, 4 points for 2.5–2.9 g/dL, and 6 points for <2.5 g/dL. Total cholesterol scores were 0 points for ≥180 mg/dL, 1 point for 140–179 mg/dL, 2 points for 100–139 mg/dL, and 3 points for <100 mg/dL. Total lymphocyte count scores were 0 points for ≥1,600/mm^3^, 1 point for 1,200–1,599/mm^3^, 2 points for 800–1,199/mm^3^, and 3 points for <800/mm^3^. The total CONUT score (range, 0–12) was the sum of the 3 subscores. Higher scores indicate worse nutritional status: 0–1, normal; 2–4, mild malnutrition; 5–8, moderate malnutrition; and 9–12, severe malnutrition. Geriatric nutritional risk index (GNRI) was calculated using the formula as follows: GNRI = 14.89 × serum albumin (g/dL) + 41.7 × (current body weight/ideal body weight). The weight ratio was set at 1.0 if the current weight exceeded the ideal weight. Ideal body weight was calculated using the formula: for men, ideal body weight = height (cm) – 100 – [(height – 150)/4]; for women, ideal body weight = height (cm) – 100 – [(height – 150)/2.5]. GNRI scores were categorized as the following: >98, no risk; 92–98, mild risk; 82–91, moderate risk; and <82, severe risk.

### 2.3 Definition of osteoporosis, FI, and FISI

According to the World Health Organization criteria, osteoporosis was diagnosed based on bone mineral density (BMD) assessment using dual-energy x-ray absorptiometry (DXA) at the lumbar spine, femoral neck, or total hip. According to this classification, osteoporosis is diagnosed when the T-score, representing the number of standard deviations the BMD deviates from the mean of young healthy adults, is −2.5 or lower (19). FI and FI severity were assessed using the Birmingham Bowel and Urinary Symptoms Questionnaire (BHQ). The BHQ is a validated, standardized self-report instrument originally developed for assessing lower gastrointestinal and urinary symptoms in both clinical and research populations. Participants identified their predominant stool consistency via Bristol stool form scale cards and reported the type and frequency of accidental bowel leakage over the preceding 30 days. FI was defined as any involuntary loss of liquid or solid stool occurring at least once in the past 30 days. Participants who responded affirmatively to the corresponding BHQ item were classified as having FI. Severity of accidental bowel leakage was quantified using the fecal incontinence severity index (FISI) questionnaire (20). This instrument evaluated four distinct categories of leakage (gas, mucus, liquid, and solid stool) across six frequency gradations, from never to twice or more daily. Each category-frequency combination carries a specific weight (ranging from 0 to 19), and the aggregate FISI score is derived by summing these weighted values (21). Higher composite scores reflect greater severity of incontinence.

### 2.4 Assessment of covariates

Our study included the following sociodemographic, lifestyle, and clinical biomarker covariates. Age was recorded in years. Sex was categorized as male or female. Educational attainment was classified into three levels: less than high school, high school completion, or college or above. Body mass index (BMI) was stratified into underweight (<18.5), normal (18.5–24.9), overweight (25–29.9), or obese (≥30 kg/m^2^) (22). Tobacco and alcohol use were each categorized as never, former, or current consumption. Hypertension was ascertained based on any of the following: mean systolic blood pressure (SBP) of at least 130 mm Hg or diastolic blood pressure (DBP) of at least 80 mm Hg across three measurements, physician diagnosis, or use of antihypertensive medication (23). Diabetes was identified by a fasting glucose level of 126 mg/dL or higher, hemoglobin A1c of 6.5% or greater, clinician diagnosis, or prescription of glucose-lowering agents (24). Cardiovascular disease is defined as participants having confirmed the following diseases: coronary heart disease, cerebrovascular disease, peripheral arterial disease, aortic atherosclerosis and aneurysms, hypertensive heart disease, heart failure, rheumatic heart disease, and congenital heart anomalies (25). Chronic kidney disease (CKD) was ascertained based on either a glomerular filtration rate (GFR) below 60 mL/min/1.73 m^2^ or markers of kidney damage present for at least 3 months. These markers encompassed albuminuria, pathological sediment, tubular disorders, histologic abnormalities, or structural anomalies detected on renal imaging (26). Chronic obstructive pulmonary disease (COPD) was identified by the presence of persistent respiratory manifestations, including chronic cough, productive cough, dyspnea, or wheezing, in conjunction with post-bronchodilator spirometric evidence of airflow limitation (FEV₁/FVC < 0.70). All the above disease conditions were treated as binary variables (Yes vs. No) (27). Clinical biomarker covariates included waist circumference (WC), total cholesterol (TC), triglyceride (TG), low-density lipoprotein cholesterol (LDL), high-density lipoprotein cholesterol (HDL), glycated hemoglobin A1c (HbA1c), fasting glucose, C-reactive protein (CRP), uric acid (UA), albumin (ALB), total lymphocyte count (TLC), aspartate aminotransferase (AST), alanine aminotransferase (ALT), gamma-glutamyl transferase (GGT), and lactate dehydrogenase (LDH).

### 2.5 Statistical analysis

Baseline characteristics were compared using *t*-tests for continuous variables and *χ*^2^ tests for categorical variables. PNI was examined both as a continuous measure and categorized into quartiles (Q1–Q4). Multivariable logistic and linear regression models were fitted to evaluate relationships between PNI, FI, and FISI scores among osteoporotic participants. Three models were built, of which Model I was a crude model, Model II was adjusted for age, gender, and educational levels, and Model III was adjusted for all covariates except for CRP, GGT, ALB, and TLC. Nonlinear associations were explored via restricted cubic splines. Subgroup analyses stratified by age, sex, educational attainment, BMI, alcohol consumption, tobacco use, physical activity, hypertension, diabetes, CKD, and COPD were performed to assess the consistency of the association. Predictive performance of PNI for FI was quantified using receiver operating characteristic curves (ROC), yielding sensitivity, specificity, accuracy, positive predictive value, and negative predictive value. Comparative assessments against alternative malnutrition indices, such as GNRI and CONUT, were conducted, with incremental predictive value evaluated through net reclassification improvement (NRI) and integrated discrimination improvement (IDI). Robustness of findings was examined in multiple sensitivity analyses, including multiple imputation for handling missing data; adjusting for some potential confounding factors, such as use of medications that promote bowel incontinence, such as antibiotics and opioids; excluding individuals with a history of anorectal surgery and pelvic organ prolapse; and excluding neurological disorders (stroke, dementia, and Parkinson’s disease) and gastrointestinal diseases. Mediation effects of CRP and GGT on the relationship were investigated. Since the cross-sectional study design, the mediation effects were statistically significant but not a causal effect. All analyses were conducted using R (version 4.3.3), with statistical significance defined as a two-tailed *p* < 0.05.

## 3 Results

### 3.1 Baseline characteristics of the participants

Table 1 summarizes the baseline characteristics of the study population. Among the 10,906 individuals meeting inclusion and exclusion criteria, 53.13% (*n* = 5,794) were male, 50.30% (*n* = 5,486) had attained at least a college education, and 958 (8.78%) reported FI. Within the subset with osteoporosis, significant differences emerged across most covariates when comparing those with and without FI (*p* < 0.05).

**Table 1:** Baseline characteristics of the participants.

Variables	Total (*n* = 10,906), *n* (%) or mean (se)	FI (*n* = 958), *n* (%) or mean (se)	Normal (*n* = 9,948), *n* (%) or mean (se)	*p*-value
Age	48.67(17.50)	57.20 (16.31)	47.91 (17.40)	<0.001
Gender				<0.001
Female	5,112 (46.87)	507 (52.92)	4,605 (46.29)	
Male	5,794 (53.13)	451 (47.08)	5,343 (53.71)	
Educational levels				0.041
Less than High-school	2,826 (25.91)	281 (29.33)	2,545 (25.58)	
High school	2,594 (23.79)	221 (23.07)	2,373 (23.85)	
College or above	5,486 (50.30)	456 (47.60)	5,030 (50.57)	
BMI				<0.001
Underweight	274 (2.51)	28 (2.92)	246 (2.47)	
Normal weight	2,914 (26.72)	211 (22.02)	2,703 (27.17)	
Overweight	3,694 (33.88)	291 (30.38)	3,403 (34.21)	
Obesity	4,024 (36.89)	428 (44.68)	3,596 (36.15)	
Drinking status				<0.001
Never	8,435 (77.34)	672 (70.15)	7,763 (78.04)	
Former	2,471 (22.66)	286 (29.85)	2,185 (21.96)	
Current				
Smoking status				<0.001
Never	5,153 (47.25)	376 (39.25)	4,777 (48.02)	
Former	3,079 (28.23)	337 (35.17)	2,742 (27.56)	
Current	2,674 (24.52)	245 (25.58)	2,429 (24.42)	
Physical levels				<0.001
Vigorous level	2,684 (24.61)	182 (19.00)	2,502 (25.15)	
Middle level	2,715 (24.89)	253 (26.41)	2,462 (24.75)	
Low level	5,507 (50.50)	523 (54.59)	4,984 (50.10)	
Hypertension				<0.001
Yes	5,612 (51.46)	617 (64.41)	4,995 (50.21)	
No	5,294 (48.54)	341 (35.59)	4,953 (49.79)	
Diabetes				<0.001
Yes	1,594 (14.62)	217 (22.65)	1,377 (13.84)	
No	9,312 (85.38)	741 (77.35)	8,571 (86.16)	
Cardiovascular disease				<0.001
Yes	2,660 (24.39)	345 (36.01)	2,315 (23.27)	
No	8,246 (75.61)	613 (63.99)	7,633 (76.73)	
COPD				0.056
Yes	3,403 (31.20)	301 (31.42)	3,102 (31.18)	
No	7,503 (68.80)	657 (68.58)	6,846 (68.82)	
CKD				<0.001
Yes	2,973 (27.26)	402 (41.96)	2,571 (25.84)	
No	7,933 (72.74)	556 (58.04)	7,377 (74.16)	
FISI	6.86 (2.69)	16.3 (3.43)	3.13 (1.26)	<0.001
PNI	50.03 (4.65)	46.45 (4.13)	53.34 (5.26)	<0.001
TC (mg/dL)	219.05 (6.56)	223.26 (7.35)	216.62 (5.48)	<0.001
TG (mg/dL)	124.08 (5.64)	128.23 (6.45)	121.39 (4.54)	<0.001
HDL (mg/dL)	37.31 (4.50)	35.05 (5.32)	40.36 (3.83)	<0.001
LDL (mg/dL)	96.03 (8.04)	99.86 (9.23)	93.45 (7.46)	<0.001
HbA1c (%)	5.35 (1.03)	6.24 (1.25)	4.74 (0.89)	< 0.001
Fast glucose (mg/dL)	91.25 (6.37)	94.17 (7.35)	90.04 (5.42)	< 0.001
CRP (mg/L)	3.53 (1.01)	4.04 (1.20)	2.87 (0.82)	<0.001
UA (mg/dL)	6.08 (1.53)	6.81 (2.02)	5.29 (1.16)	<0.001
AST (U/L)	20.06 (3.12)	22.25 (3.73)	18.56 (2.78)	< 0.001
ALB (g/L)	42.54 (3.74)	41.45 (3.14)	44.04 (4.38)	< 0.001
TLC (10^9^/L)	1.52 (0.35)	1.05 (0.26)	1.83 (0.48)	<0.001
ALT (U/L)	26.15 (4.05)	28.26 (4.86)	24.14 (3.39)	< 0.001
GGT (U/L)	35.46 (3.58)	38.21 (4.26)	33.04 (3.05)	<0.001
LDH (U/L)	136.25 (7.05)	140.68 (7.73)	134.00 (6.26)	<0.001

BMI, body mass index; COPD, chronic obstructive pulmonary disease; CKD, chronic kidney disease; TC, total cholesterol; TG, triglyceride; LDL, low-density lipoprotein cholesterol; HDL, high-density lipoprotein cholesterol; LDL, low-density lipoprotein cholesterol; HbA1c, glycated hemoglobin A1c; CRP, C-reactive protein; UA, uric acid; ALB, albumin; TLC, total lymphocyte count; ALT, alanine aminotransferase; AST, aspartate aminotransferase; GGT, gamma-glutamyl transferase; LDH, lactate dehydrogenase. Categorical data are summarized as percentages, with group comparisons evaluated using *χ*^2^ tests. Continuous variables are expressed as means ± standard deviations (SDs), and differences between groups were assessed via unpaired *t*-tests. A two-sided *p*-value < 0.05 indicated statistical significance.

### 3.2 Association between PNI, FI, and FISI in individuals with osteoporosis

**Figure 2:**
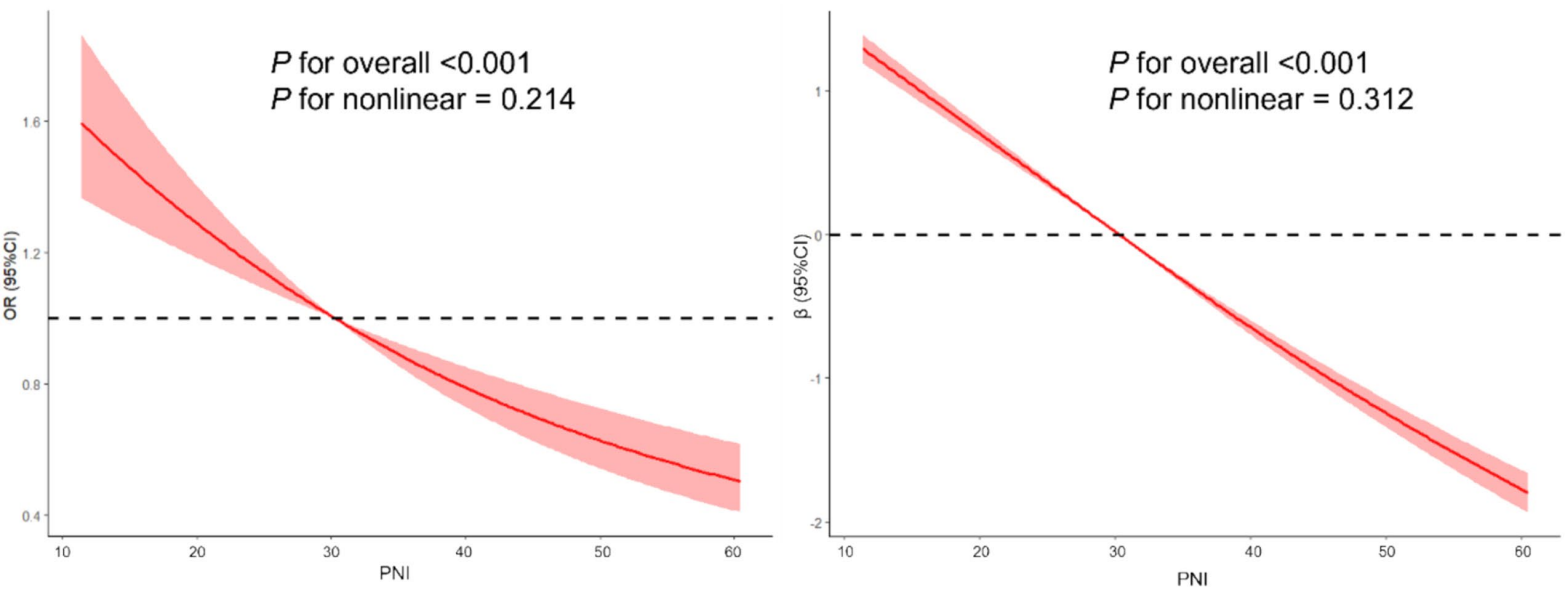
The linear associations of the PNI with FI and its severity (FISI) using RCS models. The model was adjusted for all covariates, except for CRP, GGT, ALB, and TLC. The solid lines represent the estimated odds ratios for FI and regression coefficients for FISI, with shaded bands indicating the corresponding 95% confidence intervals, based on a 3-knot spline specification. The reference value of FI risk and FISI scores was set at 32.50 and 32.03 of PNI, respectively. The plots illustrate a progressive decline in both FI risk and FISI scores as PNI increases.

Table 2 revealed negative relationships between the PNI and both FI and FISI among osteoporotic patients. Each one-unit increment in PNI was associated with 24% (Model I: odds ratio [OR], 0.76; 95% CI, 0.72–0.82), 20% (Model II: OR, 0.80; 95% CI, 0.74–0.88), and 15% (Model III: OR, 0.85; 95% CI, 0.76–0.93) lower odds of FI. When examined by quartiles, participants in the highest quartile (Q4) exhibited a 58% (Model I: OR, 0.42; 95% CI, 0.34–0.52), 52% (Model II: OR, 0.48; 95% CI, 0.40–0.59), and 43% (Model III: OR, 0.57; 95% CI, 0.48–0.75) reduction in FI risk relative to those in the lowest quartile (Q1). Similar findings were also observed for incontinence severity: each unit increase in PNI corresponded to FISI decreases of 0.65 (Model I: *β* = −0.65; 95% CI, −0.71 to −0.59), 0.55 (Model II: *β* = −0.55; 95% CI, −0.66 to −0.47), and 0.46 (Model III: *β* = −0.46; 95% CI, −0.57 to −0.38). Comparing the lowest quartile group, the highest quartile group demonstrated FISI reductions of 0.93 (Model I: *β* = −0.93; 95% CI, −1.23 to −0.62), 0.88 (Model II: *β* = −0.88; 95% CI, −1.14 to −0.57), and 0.80 (Model III: *β* = −0.80; 95% CI, −1.06 to −0.47). Trend analysis confirmed gradually decreasing relationships with the increase of PNI level across all models (all *P* for trend < 0.05). Restricted cubic splines further substantiated linear associations for both outcomes (FI: *P* for non-linear = 0.214, overall *p* < 0.001; FISI: *P* for non-linear = 0.312, overall *p* < 0.001), as depicted in Figure 2.

**Table 2:** Association of PNI with FI and FISI in individuals with osteoporosis.

Models	OR (95%CI)	*P*-value	*β* (95%CI)	*P*-value
Model I				
Continuous	0.76 (0.72, 0.82)	<0.001	−0.65 (−0.71, −0.59)	<0.001
Q1	Reference		Reference	
Q2	0.93 (0.77, 1.12)	0.482	−0.30 (−0.60, −0.01)	0.042
Q3	0.87 (0.74, 0.99)	0.048	−0.44 (−0.75, −0.14)	0.003
Q4	0.42 (0.34, 0.52)	<0.001	−0.93 (−1.23, −0.62)	<0.001
*P* for trend	0.004		<0.001	
Model II				
Continuous	0.80 (0.74, 0.88)	<0.001	−0.55 (−0.66, −0.47)	<0.001
Q1	Reference		Reference	
Q2	0.95 (0.79, 1.17)	0.446	−0.26 (−0.54, 0.07)	0.069
Q3	0.83 (0.73, 0.95)	0.040	−0.42 (−0.67, −0.05)	0.007
Q4	0.48 (0.40, 0.59)	<0.001	−0.88 (−1.14, −0.57)	<0.001
*P* for trend	0.003		0.002	
Model III				
Continuous	0.85 (0.76, 0.93)	<0.001	−0.46 (−0.57, −0.38)	<0.001
Q1	Reference		Reference	
Q2	0.97 (0.77, 1.21)	0.438	−0.20 (−0.50, 0.13)	0.154
Q3	0.88 (0.75, 1.01)	0.057	−0.35 (−0.55, −0.02)	0.011
Q4	0.57 (0.48, 0.75)	<0.001	−0.80 (−1.06, −0.47)	<0.001
*P* for trend	0.001		0.005	

Q1, quartile 1; Q2, quartile 2; Q3, quartile 3; Q4, quartile 4. Ref, reference. OR, odds ratio. Model I is a crude model. Model II adjusted for age, gender, and educational levels. Model III adjusted for all covariates, except for CRP, GGT, ALB, and TLC. *p* < 0.05 was regarded as statistically significant.

### 3.3 Subgroup analyses

In this study, subgroup analyses were performed to examine the consistency of the association between the PNI and both FI and its severity (FISI) among patients with osteoporosis. Stratification variables included age, sex, educational attainment, BMI status, alcohol and tobacco use, physical activity levels, hypertension, diabetes, cardiovascular disease, COPD, and CKD. As presented in Table 3 and Supplementary Table S1, PNI was negatively associated with FI and FISI across various subgroups (e.g., participants aged <60 years: OR, 0.78; 95% CI, 0.67–0.89; *β* = −0.44; 95% CI, −0.57 to −0.34; those aged ≥60 years: OR, 0.89; 95% CI, 0.80–0.96; *β* = −0.48; 95% CI, −0.59 to −0.38). No significant effect modification was detected for any of the stratification variables (all *P* for interaction > 0.05).

**Table 3:** Association between PNI and FI in patients with osteoporosis stratified by various subgroups.

Subgroups	OR (95%CI)	*P*-value	*P* for interaction
Age			0.346
<60	0.78 (0.67, 0.89)	<0.001	
≥60	0.89 (0.80, 0.96)	<0.001	
Gender			0.415
Female	0.82 (0.74, 0.95)	<0.001	
Male	0.86 (0.78, 0.98)	<0.001	
Educational levels			0.247
Less than High-school	0.95 (0.87, 1.05)	0.078	
High school	0.87 (0.79, 0.97)	0.002	
College or above	0.80 (0.70, 0.87)	<0.001	
BMI			0.201
Underweight	0.83 (0.76, 0.91)	<0.001	
Normal weight	0.88 (0.79, 0.99)	0.009	
Overweight	0.81 (0.71, 0.92)	<0.001	
Obesity	0.90 (0.76, 1.03)	0.085	
Drinking status			0.157
Never	0.80 (0.68, 0.95)	<0.001	
Former	0.85 (0.76, 0.96)	<0.001	
Current	0.92 (0.83, 1.04)	0.096	
Smoking status			0.124
Never	0.78 (0.66, 0.93)	<0.001	
Former	0.82 (0.74, 0.96)	<0.001	
Current	0.89 (0.78, 1.01)	0.088	
Physical levels			0.139
Vigorous	0.83 (0.74, 0.97)	<0.001	
Middle	0.87 (0.79, 0.99)	0.004	
Low	0.89 (0.80, 1.05)	0.086	
Hypertension			0.368
Yes	0.89 (0.79, 1.03)	0.074	
No	0.82 (0.73, 0.94)	<0.001	
Diabetes			0.256
Yes	0.87 (0.78, 0.98)	0.003	
No	0.81 (0.72, 0.93)	<0.001	
Cardiovascular disease			0.368
Yes	0.93 (0.81, 1.07)	0.104	
No	0.85 (0.71, 1.01)	0.057	
COPD			0.157
Yes	0.86 (0.77, 0.98)	0.006	
No	0.82 (0.73, 0.95)	0.002	
CKD			0.269
Yes	0.91 (0.82, 1.05)	0.078	
No	0.82 (0.71, 0.96)	<0.001	

BMI, body mass index; COPD, chronic obstructive pulmonary disease; CKD, chronic kidney disease; OR, odds ratio. All covariates were adjusted in the model, except for CRP, GGT, ALB, and TLC. *P* < 0.05 was regarded as statistically significant.

### 3.4 Increased predictive performance of PNI on the FI in individuals with osteoporosis

**Figure 3:**
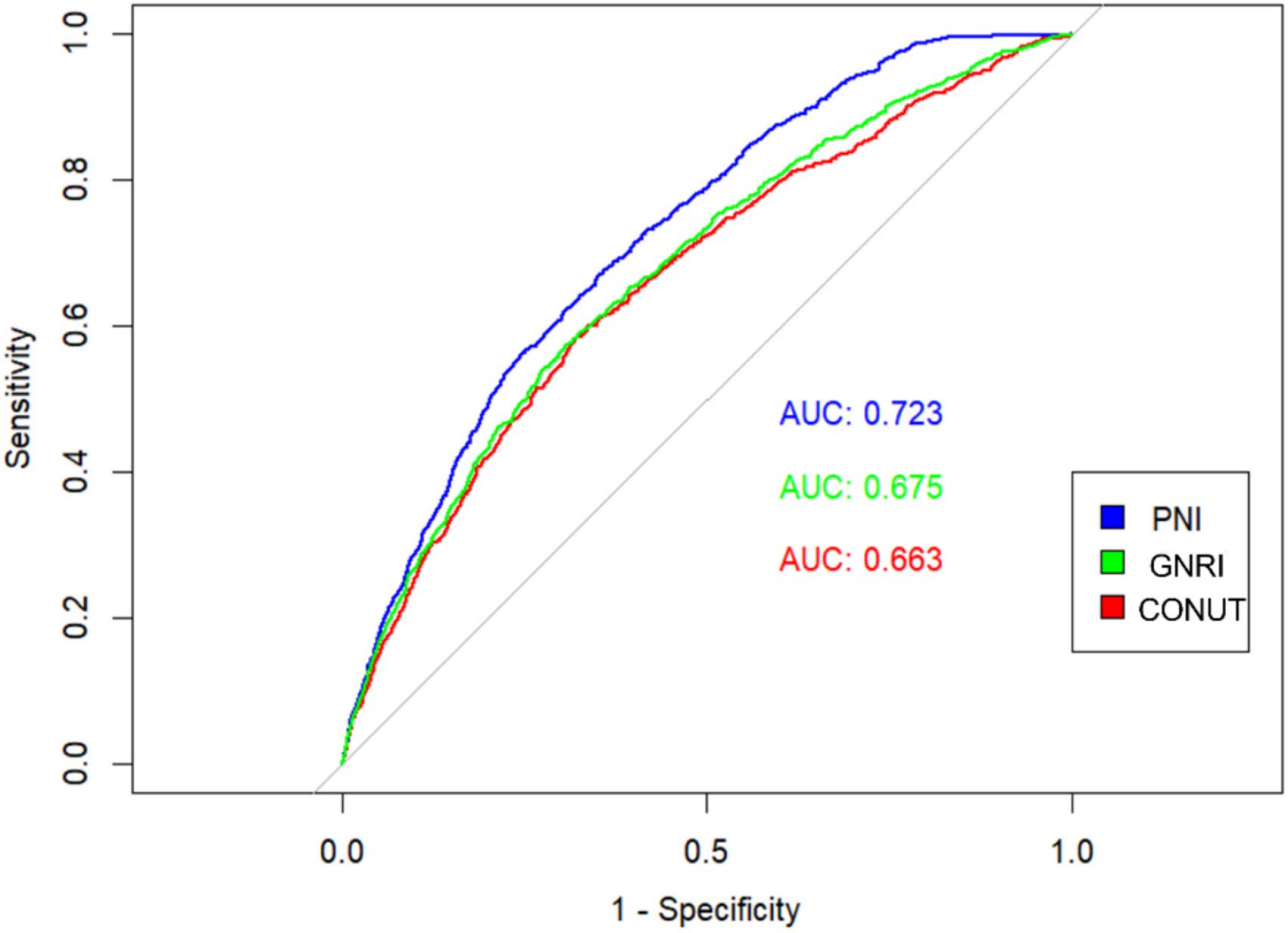
The predictive effect of some malnutrition indicators (PNI, GNRI, and CONUT) on the FI in individuals with osteoporosis. The AUC for PNI was 0.723, indicating moderate discriminative ability. For comparison, the AUC for GNRI was 0.675, and CONUT was 0.663. The model included the same covariates as in figure. The diagonal reference line represents an AUC of 0.50 (no discriminative ability). PNI showed relatively better discriminative ability compared with the other indices in this cross-sectional sample.

The predictive value of the PNI for FI was evaluated using ROC curves, as shown in Figure 3 and Supplementary Table S2. PNI demonstrated moderate discriminative ability, with an area under the curve of 0.723 (95% CI, 0.707–0.739), sensitivity of 0.668, and specificity of 0.651. Compared with alternative malnutrition indices, PNI exhibited superior predictive performance (*p* < 0.05) (Supplementary Table S3). Incremental predictive capacity was further assessed using NRI and IDI. As presented in Table 4, addition of PNI significantly improved FI risk classification (NRI, 0.13; 95% CI, 0.06–0.22; *p* < 0.001) and discrimination (IDI, 0.08; 95% CI, 0.03–0.14; *p* < 0.001), indicating its enhanced utility for identifying FI in osteoporotic patients.

**Table 4:** Discriminative performance of the diagnosis of FI in individuals with osteoporosis.

Models	NRI	*P*-value	IDI	*P*-value
Basic model	Reference		Reference	
Basic model + GNRI	0.08 (0.03, 0.15)	0.004	0.04 (0.01, 0.10)	0.008
Basic model + CONUT	0.05 (−0.01, 0.12)	0.056	0.01(−0.03, 0.05)	0.087
Basic model + PNI	0.13 (0.06, 0.22)	<0.001	0.08 (0.03, 0.14)	<0.001

NRI, net reclassification improvement; IDI, integrated discrimination improvement; GNRI, geriatric nutritional risk index; CONUT, controlling nutritional status; PNI, prognostic nutritional index. Basic model included all covariates, except for GGT, CRP, ALB, and TLC. *P* < 0.05 was regarded as statistically significant.

### 3.5 The mediation effects of the CRP and GGT

**Figure 4:**
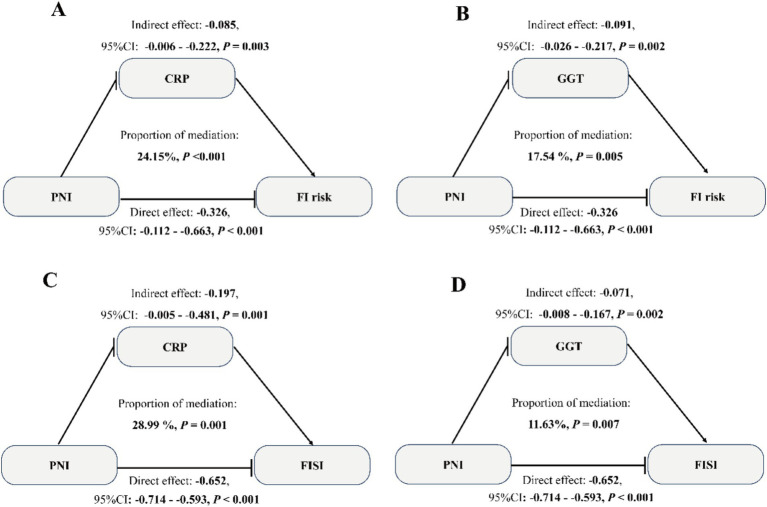
The mediating roles of CRP **(A,C)** and GGT **(B,D)** in the relationship between the PNI and both FI and its severity (FISI) among osteoporotic individuals. All covariates were adjusted in the model, except for CRP, GGT, ALB, and TLC. Solid arrows denote statistically significant pathways. The indirect effects mediated by CRP and GGT, along with the direct effect of PNI, were estimated. Both biomarkers emerged as significant mediators, accounting for a considerable portion of the total association.

Mediation analysis examined the potential mediating roles of CRP and GGT in the relationship between the PNI and both FI and its severity (FISI) in patients with osteoporosis. As shown in Supplementary Table S4, after full covariate adjustment, CRP was positively associated with FI (OR = 1.32; 95% CI, 1.21–1.55; *p* < 0.001) and FISI (*β* = 0.55; 95% CI, 0.41–0.72; *p* < 0.001). Similarly, GGT demonstrated positive associations with FI (OR = 1.44; 95% CI, 1.30–1.63; *p* < 0.001) and FISI (*β* = 0.63; 95% CI, 0.50–0.79; *p* < 0.001). Conversely, both biomarkers were negatively associated with PNI (Supplementary Table S5). After full covariate adjustment, CRP was negatively related to PNI for FI (*β* = −0.42; 95% CI, −0.56 to −0.30; *p* < 0.001) and FISI (*β* = −0.61; 95% CI, −0.78 to −0.48; *p* < 0.001), as was GGT (FI: *β* = −0.51; 95% CI, −0.69 to −0.35; *p* < 0.001; FISI: *β* = −0.70; 95% CI, −0.84 to −0.59; *p* < 0.001). In cross-sectional exploratory mediation analyses, CRP accounted for 24.15 and 28.99% of the total statistical association between PNI and FI and FISI, respectively, and GGT accounted for 17.54 and 11.63%, respectively (Figure 4). Given the cross-sectional design, these findings do not imply causality but rather suggest potential intermediate relationships that require confirmation in longitudinal studies.

### 3.6 Sensitivity analyses

Multiple sensitivity analyses were performed to assess the robustness of the associations between the PNI and both FI and its severity (FISI) in osteoporotic patients. First, multiple imputation was employed to address potential bias from missing data; the negative associations remained essentially unchanged after imputation (Supplementary Table S6). Second, to evaluate the influence of comorbid conditions, we reanalyzed the data after excluding individuals with a history of anorectal surgery, pelvic organ prolapse, neurological disorders (stroke, dementia, and Parkinson’s disease), and gastrointestinal diseases. The negative relationships persisted in our analyses (Supplementary Table S7). Third, given that certain medications (e.g., antibiotics and opioids) may promote bowel incontinence, we additionally adjusted for these agents in the regression models; the associations again remained robust (Supplementary Table S8).

## 4 Discussion

In this study, we designed a retrospective cross-sectional study to explore the association between PNI, FI, and FISI in osteoporotic patients. Our results revealed that.

The PNI was negatively associated with FI and FISI in osteoporotic patients. Furthermore, the dose–response relationships were nearly linear. In addition, the PNI had a moderate predictive effect on FI in osteoporotic patients and a higher predictive performance compared to other malnutrition indices (GNRI and CONUT). Inflammation factors (CRP) and oxidative stress markers (GGT) partially mediated the association of PNI with FI and FISI in osteoporotic patients. In subgroups and several sensitivity analyses, the negative association remained stable.

Osteoporosis is a common orthopedic condition, particularly prevalent among the elderly, which poses significant health burdens primarily through its association with fragility fractures, which markedly increase morbidity and mortality (28). Beyond fractures, the disease confers substantial complications, including chronic pain, progressive spinal deformity with height loss and kyphosis, and profound physical limitations that frequently lead to loss of independence (29, 30). Hip fractures, in particular, are associated with excess mortality, largely attributable to complications of prolonged immobilization such as venous thromboembolism, pneumonia, pressure ulcers, and muscle wasting (31). Emerging evidence also implicates osteoporosis in gastrointestinal disturbances, including constipation and, in severe cases, fecal incontinence, resulting from spinal compression-induced alterations in abdominal pressure and pelvic floor integrity (32). Collectively, these complications underscore osteoporosis as a multisystem disorder extending far beyond skeletal health. It is noteworthy that FI occurs with notable frequency among patients with osteoporosis, yet its specific risk factors remain poorly characterized. Recent evidence suggests that malnutrition may represent a common pathway linking these two conditions (33, 34). However, the relationship between nutritional status and FI in osteoporotic populations, as well as the underlying mechanisms, remains incompletely understood. Therefore, we explored the association between PNI and FI and FISI in a retrospective cross-sectional analysis.

In our study, PNI showed a significant negative association with FI and FISI in individuals with osteoporosis after adjusting for relevant covariates. And the dose–response relationship between PNI, FI, and FISI was linear. These findings suggest several potential associations that may warrant further investigation. First, PNI, as a readily accessible and low-cost biomarker, was cross-sectionally associated with FI risk in this population. If confirmed in prospective studies, this association could help inform future risk stratification strategies. Second, the observed linear pattern suggests that better nutritional status (reflected by higher PNI) may be linked to lower incontinence risk and severity. However, given the cross-sectional design, whether nutritional optimization would improve continence outcomes remains to be determined in interventional studies.

In addition, the negative associations remained consistent across all subgroups. No subgroups showed a significant modifying effect on this association. The consistency of the association enhances the generalizability of our findings. This finding suggests that the protective relationship between better nutritional status and reduced incontinence risk is robust and operates independently of demographic characteristics, lifestyle factors, or comorbid conditions. Furthermore, the absence of significant interactions implies that nutritional optimization strategies aimed at improving PNI could confer broad benefits for continence outcomes, regardless of a patient’s age, sex, body mass index, or underlying comorbidities. These findings support the integration of nutritional assessment into standard osteoporosis care as a cross-cutting intervention with the potential to mitigate incontinence risk across the entire patient spectrum. However, given that the data were derived from a single center in China, the generalizability of our findings to broader populations remains limited. Consequently, it may be premature to advocate for the widespread use of the PNI as a predictive tool for FI risk in osteoporotic patients. Future validation through multicenter studies and international external cohorts is warranted to confirm the robustness and applicability of PNI across diverse settings and populations.

In our study, the PNI was a moderated predictive effect on FI in individuals with osteoporosis. Furthermore, PNI had a greater predictive performance compared to other malnutrition indicators by ROC curves. Recent studies also demonstrated that compared with traditional logistic regression, methods of mechanism learning have some advantages in capturing nonlinear relationships and higher-order interactions without prespecification (35, 36). However, logistic regression also remains the standard approach in clinical prediction studies when sample size is moderate, interpretability is prioritized, and the goal is hypothesis testing rather than pure prediction. These findings suggest that PNI may warrant further evaluation as a potential nutritional assessment index in osteoporosis-related research. Because PNI is derived from routine laboratory data (serum albumin and lymphocyte count), it is feasible and objective, with minimal additional cost. However, given the cross-sectional design, these findings should be considered exploratory and hypothesis-generating. The moderate strength of the association also suggests that FI pathogenesis is multifactorial, and future prospective studies are needed to determine whether adding other clinical variables could improve risk prediction. Collectively, these results indicate that PNI may be a useful research variable for future investigations into comprehensive nutrition assessment in osteoporotic populations.

Some evidence has shown that inflammation factors and oxidative stress are associated with the risk of various diseases, including osteoporosis and FI (37–40). In addition, previous studies have demonstrated that elevated inflammation levels and oxidative stress of such markers as CRP, IL-6, and GGT may be associated with malnutrition status (41–43). Based on the above evidence, we speculated that inflammation and oxidative stress may have a mediating effect in the association of PNI with FI in patients with osteoporosis. However, no study has explored the role of inflammation factors and oxidative stress in the association. We explored the mediating role of inflammation levels and oxidative stress using mediation analysis in this study. The cross-sectional exploratory mediation analysis revealed that CRP and GGT partially mediated the association between the PNI and both FI and its severity (FISI) in osteoporotic patients. These findings are consistent with the hypothesis that inflammation and oxidative stress may be intermediate factors in the relationship between nutritional status and gastrointestinal outcomes. The fact that only a portion of the association was explained by CRP and GGT suggests that other factors, such as sarcopenia or altered collagen metabolism, may also be involved. However, given the cross-sectional design, these interpretations remain speculative and hypothesis-generating. Future prospective cohort studies and experimental research are needed to examine whether nutritional status influences continence in this population and through which potential pathways.

The cross-sectional design of this study offers several advantages in examining the association between PNI and FI among osteoporotic patients. First, it enabled the inclusion of a relatively large sample size, enhancing statistical power to detect significant associations while adjusting for multiple confounders. Second, the use of standardized, routinely collected laboratory data, including serum albumin and lymphocyte counts, ensured objective and reliable PNI measurement, minimizing information bias. Third, the comprehensive assessment of FI using validated instruments (FISI and BHQ) allowed for precise characterization of incontinence severity. Additionally, several sensitivity analyses strengthen the robustness of the findings. These results provide foundational epidemiological evidence supporting the role of nutritional status in gastrointestinal tract dysfunction, generating hypotheses for future prospective and mechanistic investigations.

Several limitations of this study should be acknowledged. First, the cross-sectional design precludes any causal inferences regarding the relationship between the PNI and FI in osteoporotic patients. While we observed a robust linear dose–response association, reverse causality cannot be excluded, as malnutrition may predispose individuals to FI, but severe incontinence could also lead to reduced dietary intake and subsequent nutritional decline. Second, although comprehensive adjustments were made for potential confounders, residual confounding due to unmeasured variables, such as dietary habits or psychosocial factors, may persist. Third, the assessment of FI relied on self-reported questionnaires, which may be subject to recall bias or underreporting due to social desirability, despite the use of validated instruments. Fourth, PNI was calculated from single-time-point laboratory measurements, which may not reflect long-term nutritional status or fluctuations over time. Fifth, the study population was derived from health examination attendees at a single center, who may be generally healthier, more health-conscious, and have better socioeconomic status than the broader osteoporosis population. Therefore, our findings may not be generalizable to osteoporotic patients who are hospitalized, have multiple comorbidities, or lack access to routine health examinations. In addition, the single-center design limits generalizability to other populations or healthcare settings. Sixth, while mediation analyses suggested potential mechanistic pathways involving CRP and GGT, the cross-sectional nature of these data precludes definitive conclusions about temporal relationships. Finally, although PNI demonstrated superior predictive performance compared to other malnutrition indices, its moderate predictive accuracy suggests that additional factors beyond nutritional status contribute to FI pathogenesis. Prospective cohort studies with repeated measurements and external validation in diverse populations are warranted to confirm these findings and establish causality.

## 5 Conclusion

In this cross-sectional study, PNI was independently and linearly associated with both FI and FISI in osteoporotic patients. Compared with alternative malnutrition indices (GNRI and CONUT), PNI showed a relatively stronger association with FI. In exploratory mediation analyses, CRP and GGT accounted for a portion of these statistical associations, which is consistent with the hypothesis that inflammation and oxidative stress may be intermediate factors. The consistency of the findings across prespecified subgroups and multiple sensitivity analyses supports the robustness of these cross-sectional associations. However, given the study design, these results should be interpreted as exploratory and hypothesis-generating rather than as evidence of causality or specific biological pathways.

## Data Availability

The original contributions presented in the study are included in the article/Supplementary material, further inquiries can be directed to the corresponding author.
