# Supplementary material for: Association of prognostic nutritional index with fecal incontinence and fecal incontinence severity index in individuals with osteoporosis: mediating roles of C-reactive protein and gamma-glutamyl transferase
Source: Front Nutr. 2026 Jun 9;13:1828358. doi: 10.3389/fnut.2026.1828358 (PMC13286774; doi:10.3389/fnut.2026.1828358)
Supplement: Supplementary file 1 [file Table_1.DOCX]

**Table S1.** Association between PNI and FISI in patients with osteoporosis stratified by various subgroups

|  | β (95%CI) | *P*-value | *P* for interaction |
| --- | --- | --- | --- |
| Age  < 60  ≥ 60 | -0.44 (-0.57, -0.34)  -0.48 (-0.59, -0.38) | <0.001  <0.001 | 0.374 |
| Gender  Female  Male | -0.42 (-0.51, -0.31)  -0.47 (-0.56, -0.34) | < 0.001  < 0.001 | 0.381 |
| Educational levels  Less than High-school  High school  College or above | -0.33 (-0.46, -0.24)  -0.45 (-0.57, -0.31)  -0.49 (-0.60, -0.35) | < 0.001  < 0.001  < 0.001 | 0.462 |
| BMI  Underweight  Normal weight  Overweight  Obesity | -0.37 (-0.58, -0.25)  -0.43 (-0.54, -0.29)  -0.45 (-0.56, -0.31)  -0.55 (-0.63, -0.43) | < 0.001  < 0.001  < 0.001  < 0.001 | 0.471 |
| Drinking status  Never  Former  Current | -0.45 (-0.56, -0.34)  -0.40 (-0.53, -0.38)  -0.37 (-0.48, -0.28) | < 0.001  < 0.001  < 0.001 | 0.352 |
| Smoking status  Never  Former  Current | -0.53 (-0.64, -0.40)  -0.42 (-0.55, -0.30)  -0.47 (-0,58, -0.39) | < 0.001  < 0.001  < 0.001 | 0.386 |
| Physical levels  Vigorous  Middle  low | -0.56 (-0,67, -0.43)  -0.48 (-0.59, -0.39)  -0.38 (-0.51, -0.27) | < 0.001  < 0.001  < 0.001 | 0.412 |
| Hypertension  Yes  No | -0.42 (-0.51, -0.31)  -0.50 (-0.64, -0.38) | <0.001  < 0.001 | 0.503 |
| Diabetes  Yes  No | -0.38 (-0.47, -0.27)  -0.49 (-0.62, -0.36) | < 0.001  < 0.001 | 0.439 |
| Cardiovascular disease  Yes  No | -0.36 (-0.45, -0.25)  -0.47 (-0.59, -0.32) | < 0.001  < 0.001 | 0.458 |
| COPD  Yes  No | -0.40 (-0.51, -0.31)  -0.53 (-0.64, -0.42) | <0.001  <0.001 | 0.489 |
| CKD  Yes  No | -0.42 (-0.55, -0.30)  -0.50 (-0.61, -0.41) | <0.001  <0.001 | 0.515 |

**Abbreviations:** BMI: body mass index, COPD: chronic obstructive pulmonary disease, CKD: chronic kidney disease, OR: odds ratio. Notes: All covariates were adjusted in the model, except for CRP, GGT, ALB, and TLC. *P* < 0.05 was regarded as statistically significant.

**Table S2.** The related indicator of the predictive effect of three malnutrition indices on FI risk in patients with osteoporosis

|  | PNI | GNRI | CONUT |
| --- | --- | --- | --- |
| AUC (95%CI) | 0.723(0.707-0.739) | 0.675(0.657-0.693) | 0.663(0.645-0.682) |
| Sensitivity | 0.668 | 0.575 | 0.584 |
| Specificity | 0.651 | 0.693 | 0.679 |
| Accuracy | 0.659 | 0.634 | 0.631 |
| PPV | 0.662 | 0.622 | 0.621 |
| NPV | 0.665 | 0.611 | 0.613 |

**Abbreviations:** PNI: prognostic nutritional index, GNRI: geriatric nutritional risk index, CONUT:

controlling nutritional status, PPV: positive predictive value, NPV: negative predictive value. **Notes:** Basic model included all covariates, except for GGT, CRP, ALB, and TLC. *P* < 0.05 was regarded as statistically significant.

**Table S3.** Comparative analysis of three malnutrition indices

|  | PNI vs CONUT |  | PNI vs GNRI |  | GNRI vs CONUT |  |
| --- | --- | --- | --- | --- | --- | --- |
|  | Difference | *P-* value | Difference | *P*- value | Difference | *P*- value |
| FI  C-statistic (95%CI) | 0.061 (0.044, 0.072) | <0.001 | 0.052 (0.038, 0.057) | <0.001 | 0.009 (0.003, 0.021) | 0.007 |

**Abbreviations:** PNI: prognostic nutritional index, GNRI: geriatric nutritional risk index, CONUT: controlling nutritional status. **Notes:** The model included all covariates, except for GGT, CRP, ALB, and TLC. *P* < 0.05 was regarded as statistically significant.

**Table S4.** Association of CRP and GGT with FI and FISI in patients with osteoporosis

|  | FI | | FISI | |  |
| --- | --- | --- | --- | --- | --- |
|  | OR (95%CI) | *P*-value | β (95%CI) | *P*-value | |
| CRP |  |  |  |  | |
| Continuous | 1.32 (1.21, 1.55) | < 0.001 | 0.55 (0.41, 0.72) | < 0.001 | |
| Q1 | Reference |  | Reference |  | |
| Q2 | 1.03 (0.94, 1.14) | 0.073 | 0.06 (-0.04, 0.16) | 0.076 | |
| Q3 | 1.15 (1.04, 1.30) | 0.010 | 0.20 (0.06, 0.42) | 0.005 | |
| Q4 | 1.41 (1.24, 1.63) | <0.001 | 0.64 (0.45, 0.85) | < 0.001 | |
| *P* for trend | 0.003 |  | 0.005 |  | |
| GGT |  |  |  |  | |
| Continuous | 1.44 (1.30, 1.63) | < 0.001 | 0.63 (0.50, 0.79) | < 0.001 | |
| Q1 | Reference |  | Reference |  | |
| Q2 | 1.10 (0.99, 1.21) | 0.051 | 0.19 (0.06, 0.27) | 0.002 | |
| Q3 | 1.23 (1.12, 1.37) | <0.001 | 0.33 (0.22, 0.49) | < 0.001 | |
| Q4 | 1.52 (1.31, 1.79) | <0.001 | 0.73 (0.54, 0.89) | < 0.001 | |
| *P* for trend | 0.002 |  | 0.001 |  | |

**Abbreviations:** CRP: C-reactive protein, GGT: gamma-glutamyl transferase. **Notes:** The model included all covariates, except for GGT, CRP, ALB, and TLC. *P* < 0.05 was regarded as statistically significant.

**Table S5.** Association of CRP and GGT with PNI in patients with osteoporosis

|  | FI | | FISI | |
| --- | --- | --- | --- | --- |
|  | β (95%CI) | *P*-value | β (95%CI) | *P*-value |
| CRP |  |  |  |  |
| Continuous | -0.42 (-0.56, -0.30) | < 0.001 | -0.61 (-0.78, - 0.48) | < 0.001 |
| Q1 | Reference |  | Reference |  |
| Q2 | -0.14 (-0.24, 0.03) | 0.073 | -0.20 (-0.33, 0.06) | 0.089 |
| Q3 | -0.35 (-0.51, -0.21) | < 0.001 | -0.45 (-0.62, -0.32) | < 0.001 |
| Q4 | -0.61 (-0.74, -0.48) | <0.001 | -0.74 (-0.89, -0.55) | < 0.001 |
| *P* for trend | 0.002 |  | 0.001 |  |
| GGT |  |  |  |  |
| Continuous | -0.51 (-0.69, -0.35) | < 0.001 | -0.70 (-0.84, -0.59) | < 0.001 |
| Q1 | Reference |  | Reference |  |
| Q2 | -0.20 (-0.39, -0.03) | 0.037 | -0.29 (-0.43, -0.17) | 0.002 |
| Q3 | -0.46 (-0.62, -0.29) | <0.001 | -0.53 (-0.72, -0.39) | < 0.001 |
| Q4 | -0.72 (-0.85, -0.56) | <0.001 | -0.73 (-0.89, -0.58) | < 0.001 |
| *P* for trend | 0.001 |  | < 0.001 |  |

**Abbreviations:** CRP: C-reactive protein, GGT: gamma-glutamyl transferase. **Notes:** The model included all covariates, except for GGT, CRP, ALB, and TLC. *P* < 0.05 was regarded as statistically significant.

**Table S6.** Association of PNI with FISI and FI in patients with osteoporosis after imputation of missing data

|  | OR (95%CI) | *P*-value | β (95%CI) | *P*-value |
| --- | --- | --- | --- | --- |
| Model Ⅰ |  |  |  |  |
| Continuous | 0.79 (0.73, 0.86) | < 0.001 | -0.58 (-0.70, -0.51) | <0.001 |
| Q1 | Reference |  | Reference |  |
| Q2 | 0.96 (0.81, 1.17) | 0.523 | -0.26 (-0.53, 0.02) | 0.063 |
| Q3 | 0.84 (0.76, 0.96) | 0.011 | -0.39 (-0.68, -0.10) | 0.003 |
| Q4 | 0.45 (0.34, 0.58) | < 0.001 | -0.86 (-1.16, -0.43) | < 0.001 |
| *P* for trend | 0.003 |  | 0.003 |  |
| Model Ⅱ |  |  |  |  |
| Continuous | 0.83 (0.77, 0.93) | < 0.001 | -0.51 (-0.63, -0.42) | < 0.001 |
| Q1 | Reference |  | Reference |  |
| Q2 | 0.98 (0.78, 1.21) | 0.446 | -0.20 (-0.44, 0.07) | 0.089 |
| Q3 | 0.86 (0.76, 0.98) | 0.040 | -0.32 (-0.56, -0.04) | 0.007 |
| Q4 | 0.48 (0.38, 0.63) | < 0.001 | -0.78 (-1.04, -0.50) | < 0.001 |
| *P* for trend | 0.001 |  | 0.005 |  |
| Model Ⅲ |  |  |  |  |
| Continuous | 0.86 (0.74, 0.97) | < 0.001 | -0.42 (-0.51, -0.34) | < 0.001 |
| Q1 | Reference |  | Reference |  |
| Q2 | 1.02 (0.86, 1.25) | 0.613 | -0.15 (-0.39, 0.17) | 0.154 |
| Q3 | 0.91 (0.80, 1.04) | 0.076 | -0.25 (-0.50, 0.02) | 0.065 |
| Q4 | 0.55 (0.44, 0.72) | < 0.001 | -0.65 (-0.86, -0.38) | < 0.001 |
| *P* for trend | 0.004 |  | 0.014 |  |

**Abbreviations:** Q1: quartile 1; Q2: quartile 2; Q3: quartile 3; Q4: quartile 4. *Ref*: reference. OR: odds ratio. **Notes:** Model Ⅰ is a crude model. Model II adjusted for age, gender, educational levels, and PIR. Model Ⅲ adjusted for all covariates, except for CRP, GGT, ALB, and TLC. *P* < 0.05 was regarded as statistically significant.

**Table S7.** Association of PNI with FISI and FI in patients with osteoporosis after excluding individuals with a history of anorectal surgery, pelvic organ prolapse, neurological disorders, and gastrointestinal diseases.

|  | OR (95%CI) | *P*-value | β (95%CI) | *P*-value |
| --- | --- | --- | --- | --- |
| Model Ⅰ |  |  |  |  |
| Continuous | 0.82 (0.74, 0.89) | < 0.001 | -0.50 (-0.65, -0.38) | <0.001 |
| Q1 | Reference |  | Reference |  |
| Q2 | 0.96 (0.80, 1.17) | 0.532 | -0.22 (-0.48, 0.04) | 0.073 |
| Q3 | 0.89 (0.76, 1.02) | 0.059 | -0.35 (-0.60, -0.20) | < 0.001 |
| Q4 | 0.50 (0.38, 0.64) | < 0.001 | -0.74 (-1.06, -0.38) | < 0.001 |
| *P* for trend | 0.006 |  | 0.005 |  |
| Model Ⅱ |  |  |  |  |
| Continuous | 0.86 (0.77, 0.97) | 0.001 | -0.46 (-0.58, -0.33) | < 0.001 |
| Q1 | Reference |  | Reference |  |
| Q2 | 0.98 (0.83, 1.21) | 0.489 | -0.14 (-0.38, 0.10) | 0.098 |
| Q3 | 0.87 (0.79, 0.98) | 0.044 | -0.28 (-0.50, -0.01) | 0.045 |
| Q4 | 0.56 (0.41, 0.70) | < 0.001 | -0.67 (-0.94, -0.32) | < 0.001 |
| *P* for trend | 0.002 |  | 0.008 |  |
| Model Ⅲ |  |  |  |  |
| Continuous | 0.89 (0.78, 0.99) | 0.015 | -0.36 (-0.50, -0.24) | < 0.001 |
| Q1 | Reference |  | Reference |  |
| Q2 | 1.01 (0.86, 1.24) | 0.615 | -0.08 (-0.29, 0.21) | 0.216 |
| Q3 | 0.92 (0.81, 1.05) | 0.067 | -0.20 (-0.45, 0.05) | 0.105 |
| Q4 | 0.61 (0.44, 0.73) | < 0.001 | -0.60 (-0.80, -0.28) | < 0.001 |
| *P* for trend | 0.007 |  | 0.028 |  |

**Abbreviations:** Q1: quartile 1; Q2: quartile 2; Q3: quartile 3; Q4: quartile 4. *Ref*: reference. OR: odds ratio. **Notes:** Model Ⅰ is a crude model. Model II adjusted for age, gender, educational levels, and PIR. Model Ⅲ adjusted for all covariates, except for CRP, GGT, ALB, and TLC. *P* < 0.05 was regarded as statistically significant.

**Table S8.** Association of PNI with FISI and FI in patients with osteoporosis after additionally adding some potential confounding factors, such as certain medications (e.g., antibiotics and opioids)

|  | OR (95%CI) | *P*-value | β (95%CI) | *P*-value |
| --- | --- | --- | --- | --- |
| Model Ⅰ |  |  |  |  |
| Continuous | 0.74 (0.67, 0.83) | < 0.001 | -0.60 (-0.78, -0.41) | <0.001 |
| Q1 | Reference |  | Reference |  |
| Q2 | 0.90 (0.76, 1.08) | 0.536 | -0.26 (-0.51, -0.08) | 0.073 |
| Q3 | 0.78 (0.67, 0.92) | 0.007 | -0.40 (-0.65, -0.25) | < 0.001 |
| Q4 | 0.54 (0.40, 0.68) | < 0.001 | -0.78 (-1.09, -0.46) | < 0.001 |
| *P* for trend | 0.002 |  | 0.001 |  |
| Model Ⅱ |  |  |  |  |
| Continuous | 0.80 (0.70, 0.93) | 0.001 | -0.53 (-0.69, -0.43) | < 0.001 |
| Q1 | Reference |  | Reference |  |
| Q2 | 0.92 (0.83, 1.14) | 0.468 | -0.20 (-0.43, 0.02) | 0.072 |
| Q3 | 0.83 (0.72, 0.94) | 0.002 | -0.32 (-0.54, -0.13) | < 0.001 |
| Q4 | 0.56 (0.45, 0.74) | < 0.001 | -0.70 (-0.98, -0.37) | < 0.001 |
| *P* for trend | 0.001 |  | 0.002 |  |
| Model Ⅲ |  |  |  |  |
| Continuous | 0.86 (0.76, 0.97) | 0.015 | -0.45 (-0.57, -0.26) | < 0.001 |
| Q1 | Reference |  | Reference |  |
| Q2 | 0.95 (0.87, 1.18) | 0.626 | -0.12 (-0.31, 0.08) | 0.135 |
| Q3 | 0.86 (0.75, 0.98) | 0.042 | -0.22 (-0.43, -0.04) | 0.008 |
| Q4 | 0.63 (0.49, 0.80) | < 0.001 | -0.63 (-0.85, -0.35) | < 0.001 |
| *P* for trend | 0.004 |  | 0.003 |  |

**Abbreviations:** Q1: quartile 1; Q2: quartile 2; Q3: quartile 3; Q4: quartile 4. *Ref*: reference. OR: odds ratio. **Notes:** Model Ⅰ is a crude model. Model II adjusted for age, gender, educational levels, and PIR. Model Ⅲ adjusted for all covariates, except for CRP, GGT, ALB, and TLC. *P* < 0.05 was regarded as statistically significant.
